# Supplementary material for: The association between menstrual hygiene, workplace sanitation practices and self-reported urogenital symptoms in a cross-sectional survey of women working in Mukono District, Uganda
Source: PLoS One. 2023 Jul 20;18(7):e0288942. doi: 10.1371/journal.pone.0288942 (PMC10358934; doi:10.1371/journal.pone.0288942)
Supplement: S1 File — (DOCX) [file pone.0288942.s001.docx]

# S1. Supplementary Materials

## Table 1

| **Questions from the women and workplaces survey included in study analyses** | |
| --- | --- |
| **Workplace Type** | Marketplace; Health Care Facility; School |
| **Age** | How old are you? |
| **School** | What is the highest level of school you attended? |
| **A1.1, A1.2, A1.3, A1.4, A1.5** | Over the past 12 months how often, if ever, have you or anyone in your household gone without enough food to eat?  Over the past 12 months how often, if ever, have you or anyone in your household gone without enough clean water for home use?  Over the past 12 months how often, if ever, have you or anyone in your household… Gone without medicines or medical treatment?  Over the past 12 months how often, if ever, have you or anyone in your household… Gone without enough fuel to cook your food?  Over the past 12 months how often, if ever, have you or anyone in your household… Gone without a cash income? |
| **MPQ1** | What were all of the menstrual materials [things] you used when you were at home during your last period? |
| **MPQ6** | What were all of the menstrual materials you used when you were at work during your last period? |
| **MPQ3** | During your last menstrual period, how many times did you change your menstrual materials on the heaviest day? |
| **MPN16** | When at home during your last period… Were you able to change your menstrual materials when you wanted to? |
| **MPN23** | When at work during your last menstrual period… Were you able to change your menstrual materials when you wanted to? |
| **MPQ4** | Where did you most often change your menstrual materials when you were at home during your last period? |
| **MPQ5** | Where did you dispose of your used menstrual materials at home during your last period? |
| **MPQ9** | Where did you most often change your materials when you were at work during your last period? |
| **MPQ13** | How often did you wash your hands before changing your menstrual materials during your last period? |
| **MPQ15** | What was the main place you disposed of your used materials at work during your last period? |
| **MPQ16** | Did you wash and reuse any menstrual materials/things during your last period? |
| **MPQ17** | How often did you use soap or detergent to soak or wash your menstrual materials during your last period? |
| **MPQ18** | During your last period, how often did you dry your materials in the sun? |
| **MPQ21** | During your last period, did you iron your menstrual materials before you reused them? |
| **MPQ19** | When your menstrual materials were drying, did you cover them with anything? |
| **MPQ20** | How often, during your last period were your menstrual materials completely dry before you used them? |
| **C2** | What place do you usually use to make a short call [urinate] when you are at work? |
| **C7** | I’d like you to tell me yes or no if these are present at your [short call] urination place: Water for washing available at the location |
| **C8** | Do you usually take anything with you to the place you make short calls? Own water for cleansing |
| **SC1** | Over the last 30 days did you need to delay making a short call [urinating] at work? |
| **F0** | How is your health in general? |
| **F1** | Burning or discomfort when urinating |
| **F2** | Itching or burning in your genital region |
| **F3** | Unpleasant or fishy odour from genital area |
| **F4** | Abnormal vaginal discharge (unusual texture and color: e.g., milky, white, grey, green or yellow discharge) |
| **F5** | Did you discuss your symptoms with a health care provider when they occurred? |

## Table 2

| **Materials used at home and work during last period** | **% (n)** |
| --- | --- |
| Disposable pad | 63.9 (319) |
| Cloth/towel | 14.8 (74) |
| Reusable pad | 5.8 (29) |
| Disposable pad & cloth/towel | 5.7 (28) |
| Disposable pad & reusable pad | 3.0 (15) |
| Underwear alone | 1.4 (7) |
| Cotton wool | 0.8 (4) |
| Disposable pad & cotton wool | 0.8 (4) |
| Cotton wool & gauze | 0.8 (4) |
| Disposable pad & cotton wool & gauze | 0.6 (3) |
| Disposable pad & underwear alone | 0.4 (2) |
| Disposable pad & toilet paper | 0.4 (2) |
| Cloth/towel & toilet paper | 0.4 (2) |
| Cloth/towel & cotton wool | 0.4 (2) |
| Toilet paper | 0.4 (2) |
| Cloth/towel & toilet paper & cotton wool | 0.2 (1) |
| Disposable pad & cloth/towel & reusable pad | 0.2 (1) |
| **Total** | **499** |

## Table 3

| **Materials used** | **% (n)** |
| --- | --- |
| **Disposable pad** | **63.9 (319)** |
| **Reusable pads sterile or not reused (used with or without disposable pads)** | **7.6 (38)** |
| Reusable pad only, sterilised | 5.0 (25) |
| Reusable pad & disposable pad, sterilised | 2.2 (11) |
| Reusable pad & disposable pads, not reused | 0.4 (2) |
| **Improvised methods sterile or not reused (used with or without disposable/reusable pads)** | **22.8 (114)** |
| Cloth/towel only, sterilised | 10.4 (52) |
| Cloth/towel only, disposed | 2.2 (11) |
| Cloth/towel & disposable pad, sterilised | 3.0 (15) |
| Cloth/towel & disposable pad, disposed | 0.6 (3) |
| Underwear alone only, sterilised | 0.8 (4) |
| Underwear alone only, disposed | 0.4 (2) |
| Cotton wool only, disposed | 0.8 (4) |
| Disposable pad & cotton wool, disposed | 0.6 (3) |
| Disposable pad & cotton wool, sterilised | 0.2 (1) |
| Cotton wool & gauze, disposed | 0.8 (4) |
| Disposable pad & cotton wool & gauze, disposed | 0.6 (3) |
| Disposable pad & underwear alone, disposed | 0.2 (1) |
| Disposable pad & underwear alone, sterilised | 0.2 (1) |
| Disposable pad & toilet paper, disposed | 0.4 (2) |
| Cloth/towel & toilet paper, sterilised | 0.4 (2) |
| Cloth/towel & cotton wool, sterilised | 0.2 (1) |
| Cloth/towel & cotton wool, disposed | 0.2 (1) |
| Toilet paper, disposed | 0.4 (2) |
| Cloth/towel & toilet paper & cotton wool, disposed | 0.2 (1) |
| Disposable pad & cloth/towel & reusable pad, sterilised | 0.2 (1) |
| **Reused methods not sterile (improvised methods or reusable pads)** | **5.6 (28)** |
| Cloth/towel only, not sterilised | 2.2 (11) |
| Cloth/towel & disposable pad, not sterilised | 2.0 (10) |
| Underwear alone only, not sterilised | 0.2 (1) |
| Reusable pad only, not sterilised | 0.8 (4) |
| Reusable pad & disposable pad, not sterilised | 0.4 (2) |
| **Total** | **499** |
| *Sterile: used soap or detergent to soak or wash materials every time and materials were completely dry before using every time in the last month if washed and reused any menstrual materials during last period Improvised methods: cloth/towel, underwear alone, cotton wool, gauze, and/or toilet paper* |  |

## Table 4

| **Symptoms experienced in the last month** | | | | **n** | **% of total eligible participants** |
| --- | --- | --- | --- | --- | --- |
| **Itching/burning in the genital region** | **Burning/discomfort when urinating** | **Abnormal vaginal discharge** | **Unpleasant/fishy odour from genital area** |  |  |
| Y |  |  |  | 33 | 7% |
| Y | Y | Y | Y | 30 | 6% |
| Y | Y |  |  | 28 | 6% |
| Y | Y | Y |  | 24 | 5% |
|  | Y |  |  | 24 | 5% |
|  |  | Y |  | 18 | 4% |
| Y |  | Y | Y | 11 | 2% |
|  | Y | Y |  | 8 | 2% |
| Y |  | Y |  | 7 | 2% |
|  |  |  | Y | 6 | 1% |
| Y |  |  | Y | 5 | 1% |
|  |  | Y | Y | 4 | 1% |
| Y | Y |  | Y | 4 | 1% |
|  | Y | Y | Y | 2 | 0% |
|  | Y |  | Y | 2 | 0% |
| **153** | **131** | **109** | **68** | **206** |  |
